# Baicalein is a novel TLR4‐targeting therapeutics agent that inhibits TLR4/HIF‐1α/VEGF signaling pathway in colorectal cancer

**DOI:** 10.1002/ctm2.564

**Published:** 2021-11-04

**Authors:** Minting Chen, Keying Zhong, Jincheng Tan, Mingjing Meng, Chok Mei Liu, Baisen Chen, Chunhua Huang, Hoi Leong Xavier Wong, Zhaoxiang Bian, Tao Su, Hiu Yee Kwan

**Affiliations:** ^1^ Centre for Cancer and Inflammation Research, School of Chinese Medicine Hong Kong Baptist University Hong Kong China; ^2^ International Institute for Translational Chinese Medicine Guangzhou University of Chinese Medicine Guangzhou Guangdong China

Dear Editor,

Toll‐like receptor 4(TLR4) is a rational therapeutic target. We have discovered that baicalein directly binds to TLR4, which is a strategy to inhibit TLR4 activity. In our study, molecular docking showed that baicalein directly bound to TLR4 protein (binding energy ‐6.6 kcal/mol), which was the same as the binding energy between TLR4 and TAK242.^1^ Three‐dimensional ribbon model of the baicalein‐TLR4 complex showed that hydrogen bonds were formed between the backbone of TLR4 at PHE‐573, ALA‐572, VAL‐548, GLN‐547, VAL‐524, PHE‐500, THR‐499, VAL‐475 (Figure 1A), and the system was stable during simulation (Figure 1B). Surface plasmon resonance study showed a dose‐dependent interaction between baicalein and TLR4 protein, with the maximum response of 598 RU (Figure 1C–E). The KD for baicalein binding to TLR4 protein was 9.324 × 10^‐5^ M, suggesting a direct binding between them. Bio‐layer interferometry also showed an interaction between baicalein and TLR4 protein, the binding signal increased with increasing concentrations of baicalein (Figure 1F). The association constant for the binding was 27.3 M^–1^s^–1^, the dissociation constant was 2.14 × 10^–2^s^–1^, equilibrium dissociation constant was 7.84 × 10^–4^ M, which revealed a direct and stable binding between baicalein and TLR4 protein.

**FIGURE 1:**
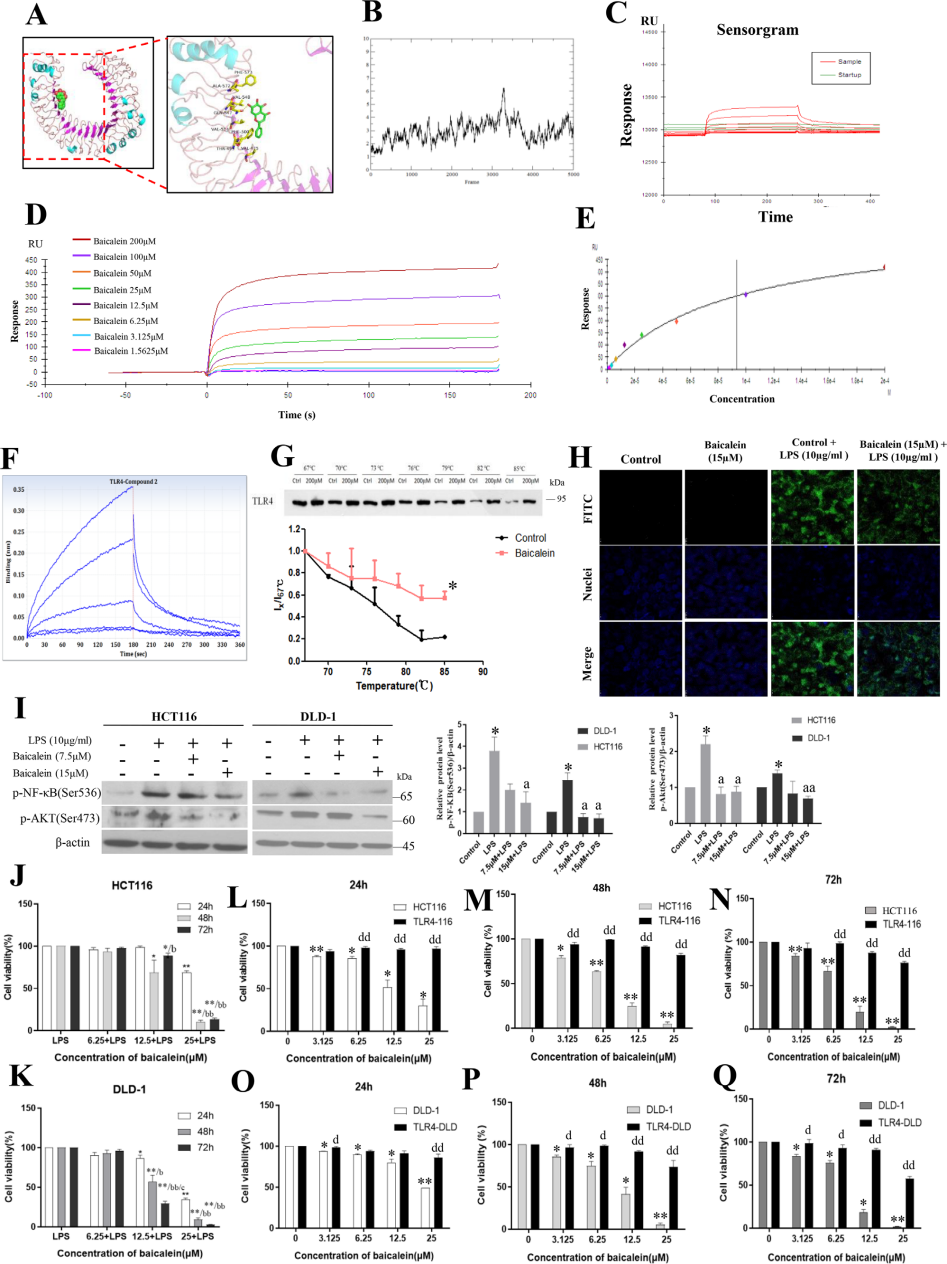
Direct physical binding of baicalein to TLR4 protein (A) Three‐dimensional ribbon model of the baicalein‐TLR4 complex. (B) Molecular dynamic and root‐mean‐square deviation values of the protein backbone showing the binding between baicalein and TLR4 during molecular stimulation. (C) Surface plasmon resonance showing the real‐time measurements of the binding affinity, (D) kinetics analysis and (E) the computer fitting of the binding of baicalein to TLR4 protein. (F) Bio‐layer interferometry analysis showing the binding between baicalein and TLR4. (G) Cellular thermal shift assay (CESTA) analysis of the binding between baicalein and TLR4 protein in CRC cells. (H) FITC‐conjugated LPS signals in TLR4‐overexpressed CRC cells. (I) Western blot showing the levels of p‐NF‐κB and p‐AKT (ser473) in the HCT116 and DLD‐1 cells. Cell viability of (J) HCT116 and (K) DLD‐1 in the presence of LPS (10μg/ml) after baicalein treatments. (L to Q) Viability of HCT116 cells, TLR4‐overexpressed HCT116 cells (TLR4‐116), DLD‐1 cells, and TLR4‐overexpressed DLD1 cells (TLR4‐DLD) after baicalein treatments. Shown is mean ± SE, n = 3 individual experiments, *P < 0.05, **P < 0.01 compared with control. a < 0.05, aa < 0.01 compared to LPS only. b < 0.05, bb < 0.01 compared with 24h and c < 0.05 compared with 48h at the indicated concentration. d < 0.05, dd < 0.01 compared to HCT116 or DLD‐1 cells under the same baicalein concentration. TLR4, Toll‐like receptor 4; LPS, lipopolysaccharides

Given the well‐known pathological roles of TLR4 in CRC,^2^ we used CRC cells as model for the study. Physical interaction between baicalein and TLR4 protein in CRC is suggested by a large thermal shift (∆*T*_m_) of TLR4 as indicated in the melting curve in the cellular thermal shift assay (Figure 1G). More importantly, binding of baicalein to TLR4 interrupted the binding of FITC‐conjugated LPS (Figure 1H), and hence inhibited TLR4 activity as demonstrated by the reduced phosphorylation of NF‐κB‐p65 and Akt upon LPS challenge (Figure 1I). Subsequently, inhibiting TLR4 activity reduced CRC cell viability (Figure 1J,K; Figure S1A,B), which was abolished when TLR4 was overexpressed in the cells (Figure S2A,B; Figure 1L‐Q). The reduced NF‐κB activity is unlikely due to TLR4, MyD88, and TIRAP because baicalein did not significantly affect their expressions (Figure S3).

We next performed drug‐targets‐disease network analysis and constructed protein‐protein interaction (PPI) network to explore the TLR4 downstream signaling molecules that mediated the inhibitory effects of baicalein. The network showed 18 yellow nodes representing the overlapped targets between CRC and baicalein, and 64 green nodes representing the target‐related pathways (Figure 2A). PPI highlighted 16 nodes representing the candidates that mediated the baicalein anti‐CRC effects (Figure 2B). Among these, hypoxia‐inducible factor‐1α (HIF‐1α) and vascular endothelial growth factor (VEGF‐A) were separately linked to 10 to 11 other proteins (Figure 2C). It has been reported that HIF‐1α and VEGF expressions are elevated in 54.93% and 56.34% of the CRC patients, respectively, and the expression levels are correlated with tumor stages and overall survival.^3^ Interestingly, our data showed that HIF‐1α and VEGF are the downstream signaling molecules of TLR4. TLR4 overexpression increased HIF‐1α and VEGF expressions in CRC cells (Figure 2D,E), whereas TLR4 knockout (Figure 2F) significantly reduced their expressions (Figure 2D). Activation of TLR4 increased HIF‐1α and VEGF expressions, which were reversed by baicalein or C34 (Figure 2G,H). Baicalein or C34 also reduced HIF‐1α and VEGF basal levels in the cells (Figure 2I,J), which was abolished when TLR4 was overexpressed (Figure 2K,L). In TLR4‐knockout cells, baicalein failed to affect HIF‐1α and VEGF expressions (Figure 2M,N).

**FIGURE 2:**
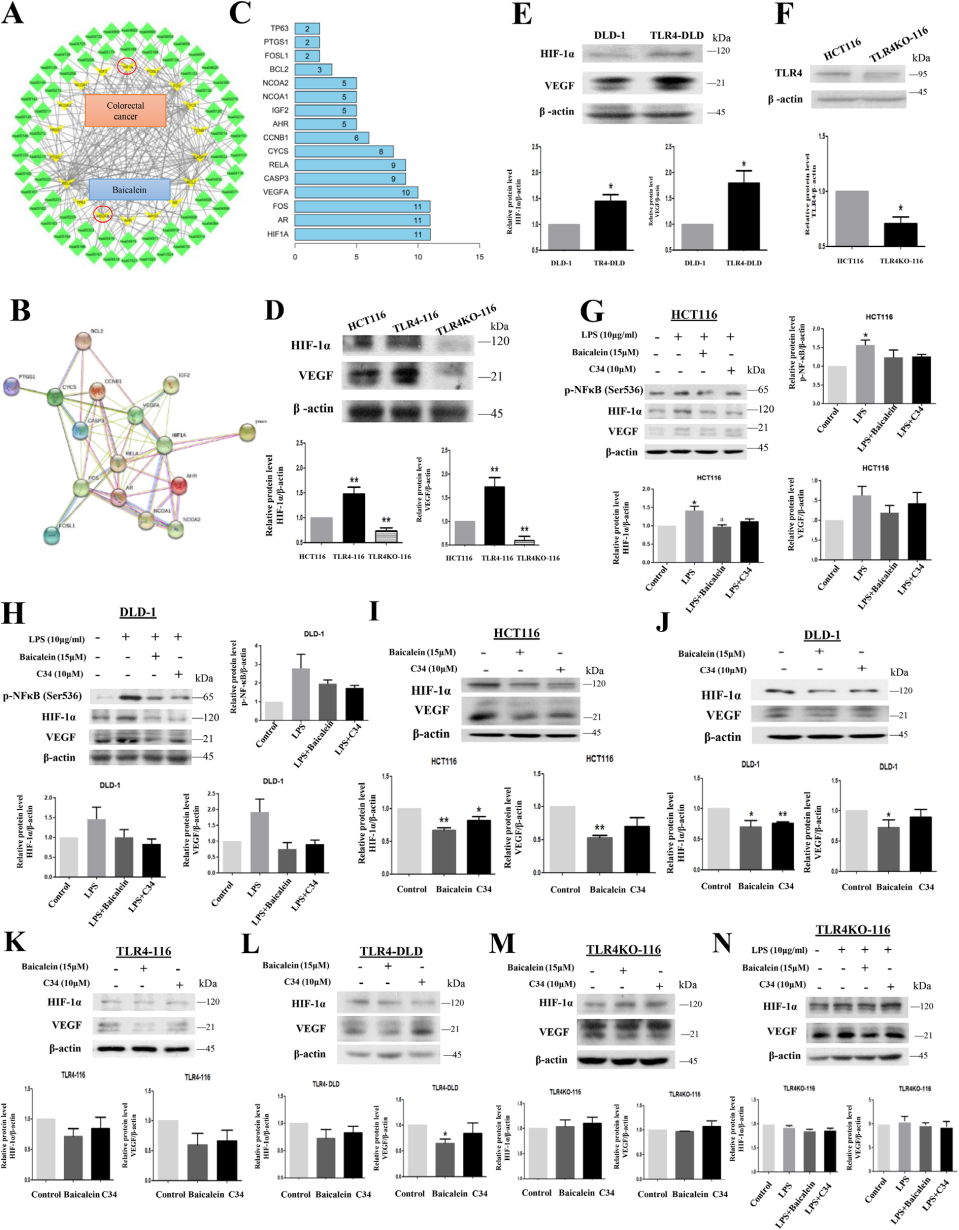
HIF‐1α and VEGF are downstream of TLR4, baicalein reduces HIF‐1α and VEGF expressions in TLR4‐dependent manner. (A) Drug–Targets–Disease network illustrates the overlapping targets between CRC and baicalein (yellow nodes) and the target‐related pathways (green nodes). (B) Protein‐protein interaction network showing the interaction between the candidate protein targets of baicalein and (C) the number of proteins interact with the highlighted protein. (F) CRISPR/Cas9‐mediated knockout of TLR4 in HCT116 cells (TLR4KO‐116). (D‐E) Protein expressions of HIF‐1α and VEGF in CRC cells (HCT116, TLR4‐overexpressed (TLR4‐116), TLR4KO‐116, DLD‐1, TLR4‐overexpressed DLD‐1 (TLR4‐DLD)). Expressions of p‐NF‐κB, HIF‐1α, and VEGF in HCT116 and DLD‐1 cells in the (G‐H) presence of LPS or (I‐J) absence of LPS after baicalein or TLR4 inhibitor C34 treatment. (K‐N) Protein expressions of HIF‐1α and VEGF in TLR4‐116, TLR4‐DLD, and TLRKO‐116 cells after baicalein or C34 treatment. Shown is mean ± SE, n = 3 individual experiments, *P < 0.05, **P < 0.01 compared to control. TLR4, toll‐like receptor 4; HIF‐1α, hypoxia‐inducible factor 1‐alpha; VEGF, vascular endothelial growth factor

HIF‐1α plays a crucial role in cancer metastasis.^4^ Our data showed that reduced HIF‐1α expression under baicalein treatment inhibited CRC cell migration, which was abolished when TLR4 was overexpressed (Figure S4).

In CRC‐bearing xenograft mouse model, baicalein significantly inhibited tumor growth, which was comparable with TAK242 (Figure 3A‐C). The treatments did not significantly affect body weight, and the blood levels of alanine aminotransferase, aspartate transaminase, creatine kinase, and urea in these mice (Figures S5), suggesting the treatments do not have apparent toxicity. Interestingly, baicalein significantly inhibited NFκB phosphorylation in the tumors (Figure 3D), the treatment did not affect NFκB and TLR4 expressions (Figure 3D,E). The reduced tumor size was likely due to the reduced cancer growth as indicated by the reduced expression of Ki67 (Figure 3F). More importantly, baicalein reduced the metastatic markers VEGF, CD31, and MMP‐2 in the tumor tissues (Figure 3G‐I). The anti‐angiogenic effect of baicalein was also demonstrated by the reduced blood vessel formation in the chick yolk sac membrane (Figure 3J), reduced vessel sprouting in the rat aortic ring model (Figure 3K), and cultured human vascular endothelial cells (Figure 3L).

**FIGURE 3:**
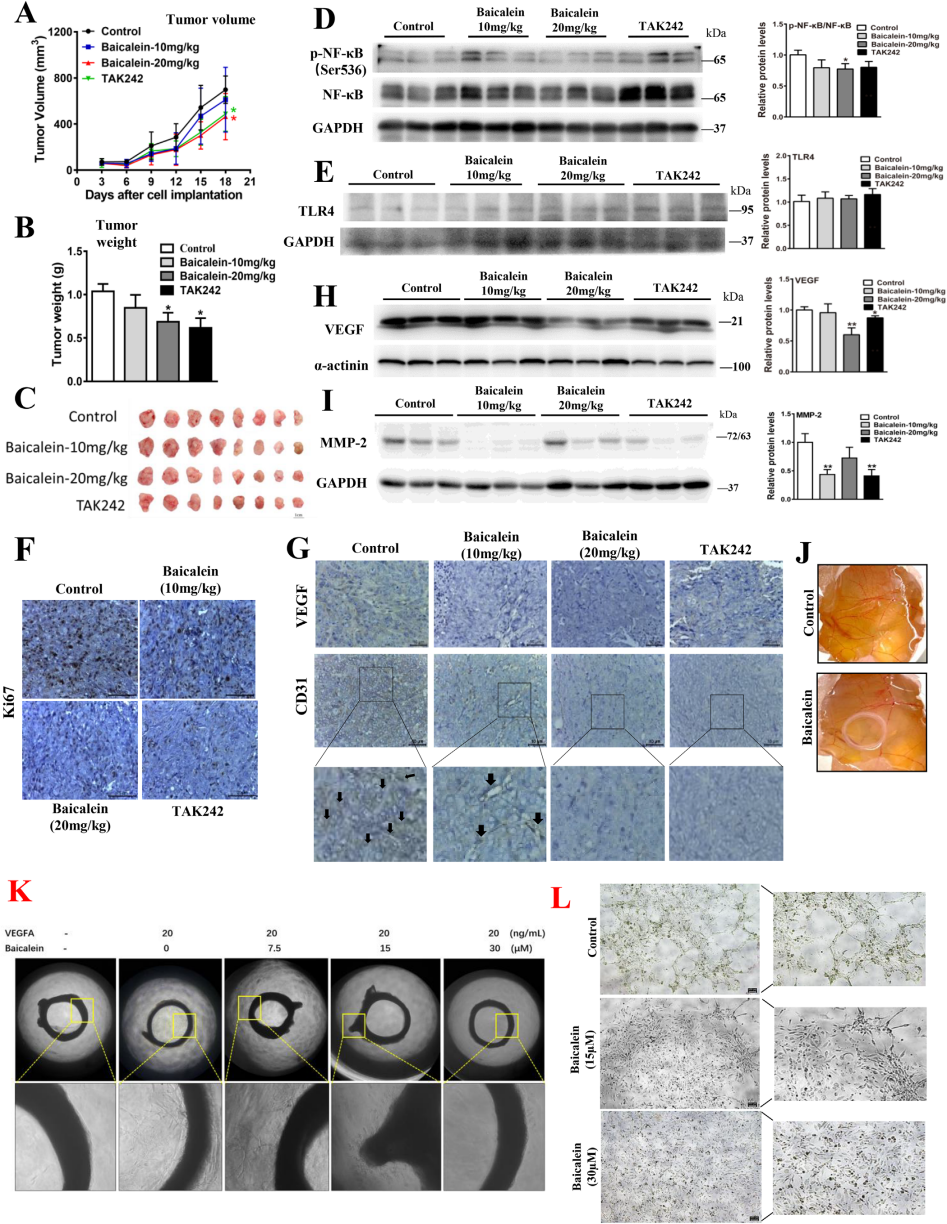
Baicalein inhibits CRC growth and metastasis via TLR4/HIF‐1α/VEGF axis in vivo. (A) Tumor volume, (B–C) tumor weight of the CRC‐bearing xenograft mouse models after baicalein treatment. Western blot showing the expressions of (D) p‐NF‐κB, NF‐κB, (E) TLR4, (H) VEGF, and (I) MMP‐2 in the xenograft tissues. Immunohistochemistry staining of (F) Ki67, (G) VEGF, and CD31 in the xenograft tissues. (J) Blood vessel formation on the chick yolk sac membrane after baicalein treatment. Vessel sprouting in (K) the rat aortic ring model of angiogenesis, and (L) tube formation of the cultured human endothelial cells after baicalein treatment. Shown is mean ± SE, n = 8 mice in each group or n = 3 individual experiments, *P < 0.05, **P < 0.01 compared to control. VEGF, vascular endothelial growth factor; MMP2, matrix metalloproteinases 2

Our data show that baicalein directly binds to TLR4, interrupts the formation of the complex (LPS.MD‐2.TLR4)_2_, and inhibits TLR4 activity, hence reduces HIF‐1α and VEGF expressions, subsequently inhibits CRC growth, angiogenesis, and reduces the cancer metastatic potential.

Metastatic CRC presents in 25% of the patients at diagnosis, one‐third of the patients experience a metastatic relapse after initial curative surgical treatment.^5^, ^6^ The binding of baicalein to TLR4 that inhibits the HIF‐1α/VEGF signaling pathway holds a promising therapy for metastatic CRC treatment. In the further, we would use target delivery system such as nanoparticles^7^ or aptamers^8^ to deliver baicalein to the tumor site that can further enhance its anti‐CRC effect.

The direct binding of baicalein to TLR4 has an added advantage in inhibiting TLR4 activity. Currently, TLR4 antagonists designed based on the modification of the lipid A4 structure,^9^ the LPS innermost region, may not have potent inhibitory effect because NMR study shows that the presence of the fatty acyl chain of the lipid A moiety is not necessary for driving TLR4 activation.^10^

In conclusion, we report the discovery that baicalein directly binds to TLR4 and inhibits TLR4/HIF‐1α/VEGF signaling pathway in CRC. Our data strongly support the translation of baicalein into novel TLR4‐targeting therapeutics for CRC treatment.

## CONFLICT OF INTEREST

The authors declare no conflict of interest.

## Supporting information


**Supplementary Figure S1 CRC cell viability after baicalein treatments**. Cell viability of **(A)** HCT116 and **(B)** DLD‐1 after baicalein treatments. Shown is mean ± SE, n = 3 individual experiments, p* < 0.05, p** < 0.01 compared with control. b < 0.05, bb < 0.01 compared with 24h and c < 0.05 compared with 48h at the indicated concentration.
**Supplementary Figure S2 Overexpression of TLR4 in CRC cells**. Western blot and quantification showing the stable overexpression of TLR4 in **(A)** HCT116 (TLR4‐116) and **(B)** DLD‐1 (TLR4‐DLD) cells. Empty vector transfection served as control. Shown is mean ± SE, n = 3 individual experiments, p* < 0.05, p** < 0.01 compared with control. TLR4, toll like receptor 4.
**Supplementary Figure S3 Expression of TLR4, MyD88 and TIRAP in CRC cells after baicalein treatments**. Western blot and quantification showing the expressions of TLR4, MyD88, TIRAP in the HCT116 and DLD‐1 cells after baicalein treatments. Shown is mean SE, n = 3 individual experiments. TLR4, toll like receptor 4; MyD88, myeloid differentiation factor 88; TIRAP, TIR domain‐containing adaptor protein.
**Supplementary Figure S4 Baicalein reduces CRC cell migration in TLR4‐dependent manner. (A‐B)** A single scratch was made in the confluent monolayer of HCT116 or TLR4‐overexpressed HCT116 (TLR4‐116) cells, or **(C‐D)** DLD‐1 or TLR4‐overexpressed DLD (TLR4‐DLD) cells. Cell migration was examined in the presence or absence of biacalein, with the presence of mitomycin C in the culture medium. The scratch was photographed at 0h, 48h and 72h after treating baicalein treatments. The relative migrated areas are analyzed by Image J software (right panel). Shown is mean ± SE, n = 3 individual experiments, p* < 0.05, p** < 0.01 as indicated.
**Supplementary Figure S5 Baicalein has no apparent toxicity to the mice. (A)** Body weight, and the blood levels of **(B)** alanine aminotransferase (ALT), **(C)** aspartate transaminase (AST), **(D)** creatine kinase (CK) and **(E)** urea in the CRC‐bearing xenograft mouse model. Shown is mean ± SE, n = 8 mice in each group.
