# Supplementary figures and images for: Baicalein is a novel TLR4‐targeting therapeutics agent that inhibits TLR4/HIF‐1α/VEGF signaling pathway in colorectal cancer
Source: Clin Transl Med. 2021 Nov 4;11(11):e564. doi: 10.1002/ctm2.564 (PMC8567042; doi:10.1002/ctm2.564)

**A**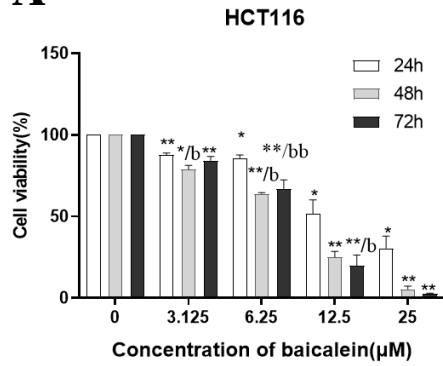**B**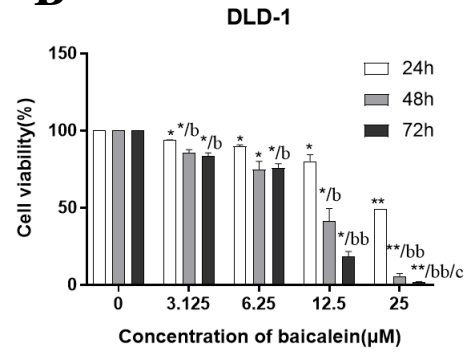

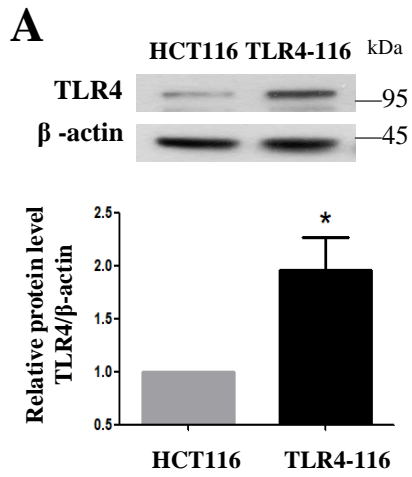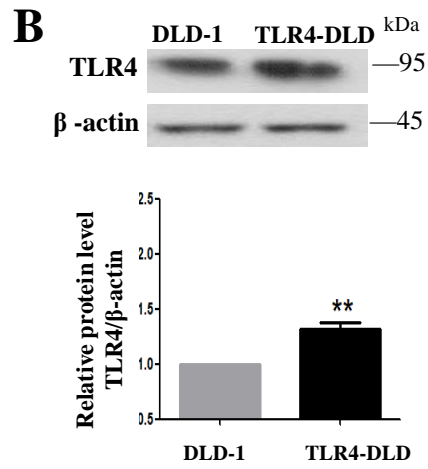

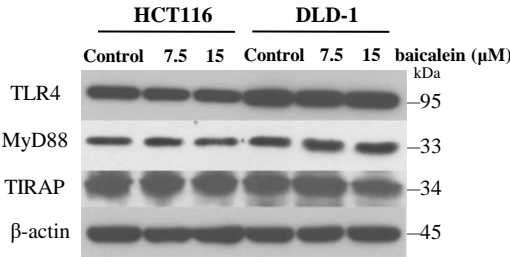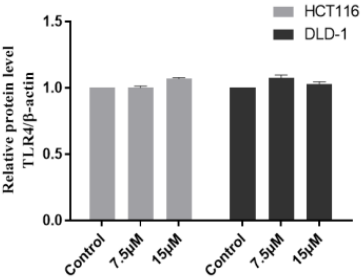

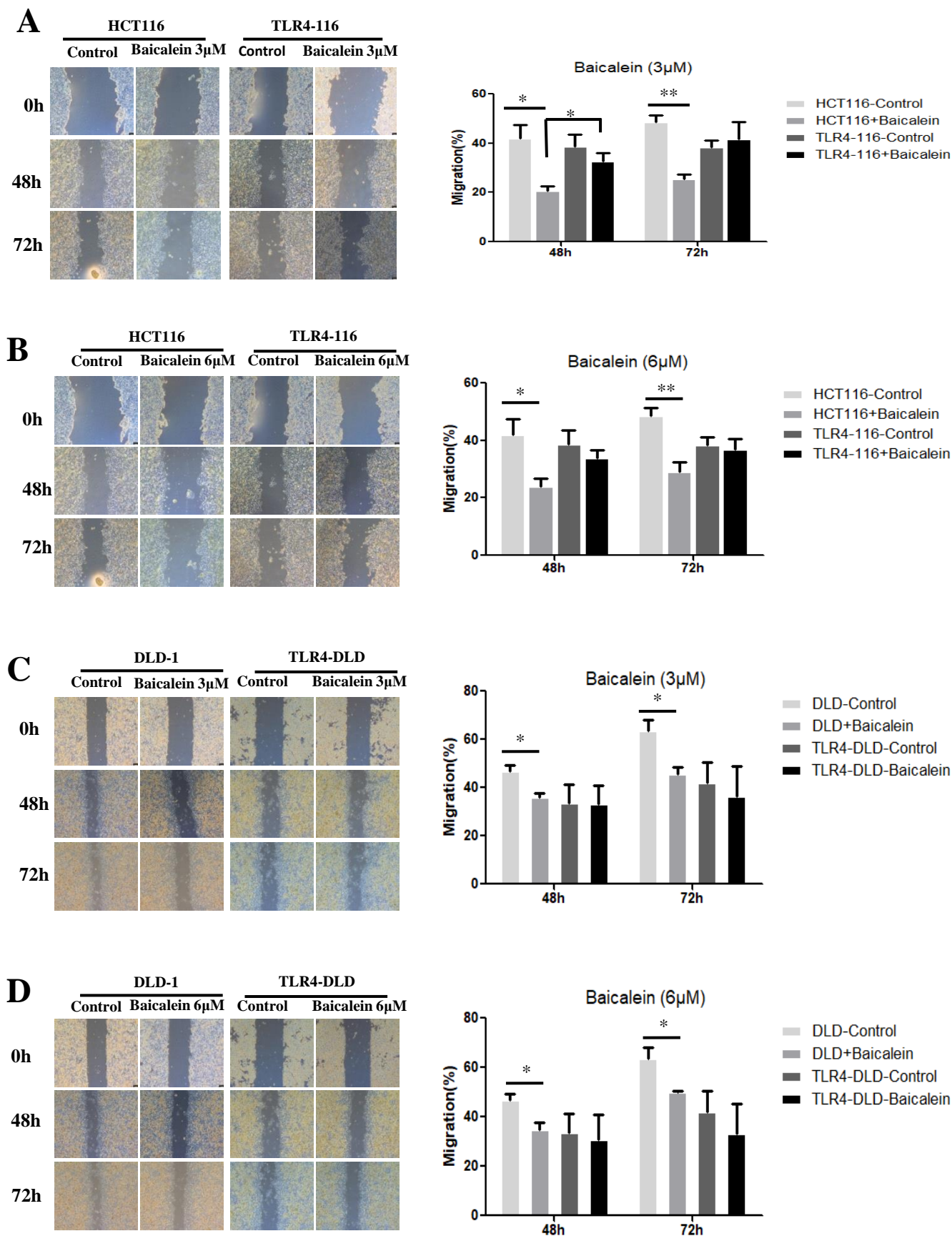

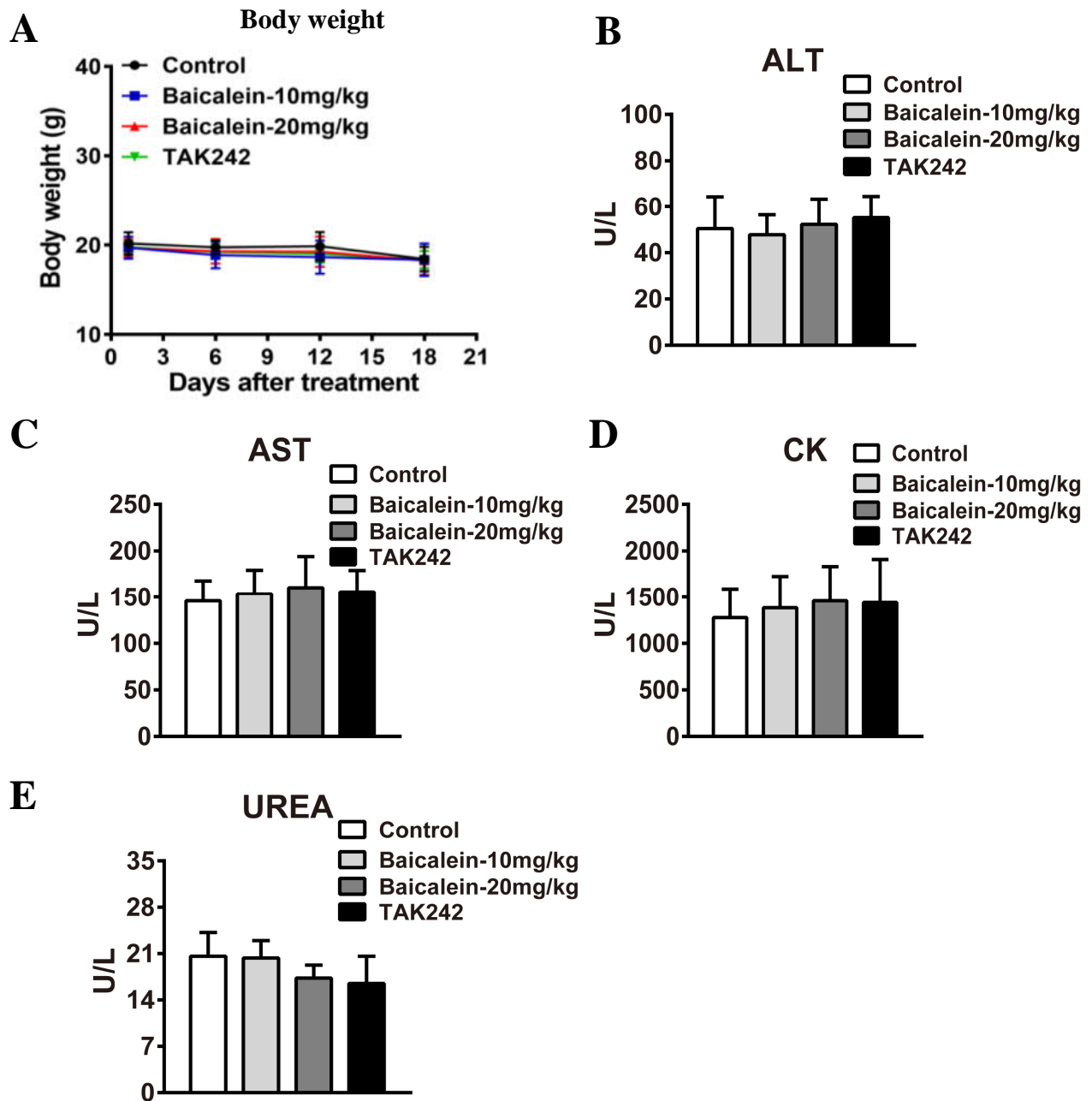

Supplement: Supplementary file 1 — Supplementary Figure S1 CRC cell viability after baicalein treatments. Cell viability of (A) HCT116 and (B) DLD‐1 after baicalein treatments. Shown is mean ± SE, n = 3 individual experiments, p* < 0.05, p** < 0.01 compared with control. b < 0.05, bb < 0.01 compared with 24h and c < 0.05 compared with 48h at the indicated concentration. Supplementary Figure S2 Overexpression of TLR4 in CRC cells. Western blot and quantification showing the stable overexpression of TLR4 in (A) HCT116 (TLR4‐116) and (B) DLD‐1 (TLR4‐DLD) cells. Empty vector transfection served as control. Shown is mean ± SE, n = 3 individual experiments, p* < 0.05, p** < 0.01 compared with control. TLR4, toll like receptor 4. Supplementary Figure S3 Expression of TLR4, MyD88 and TIRAP in CRC cells after baicalein treatments. Western blot and quantification showing the expressions of TLR4, MyD88, TIRAP in the HCT116 and DLD‐1 cells after baicalein treatments. Shown is mean SE, n = 3 individual experiments. TLR4, toll like receptor 4; MyD88, myeloid differentiation factor 88; TIRAP, TIR domain‐containing adaptor protein. Supplementary Figure S4 Baicalein reduces CRC cell migration in TLR4‐dependent manner. (A‐B) A single scratch was made in the confluent monolayer of HCT116 or TLR4‐overexpressed HCT116 (TLR4‐116) cells, or (C‐D) DLD‐1 or TLR4‐overexpressed DLD (TLR4‐DLD) cells. Cell migration was examined in the presence or absence of biacalein, with the presence of mitomycin C in the culture medium. The scratch was photographed at 0h, 48h and 72h after treating baicalein treatments. The relative migrated areas are analyzed by Image J software (right panel). Shown is mean ± SE, n = 3 individual experiments, p* < 0.05, p** < 0.01 as indicated. Supplementary Figure S5 Baicalein has no apparent toxicity to the mice. (A) Body weight, and the blood levels of (B) alanine aminotransferase (ALT), (C) aspartate transaminase (AST), (D) creatine kinase (CK) and (E) urea in the CRC‐bearing xenograft mouse model. Show [file CTM2-11-e564-s001.pdf]
